# Treatment with Hizentra in patients with primary and secondary immunodeficiencies: a real-life, non-interventional trial

**DOI:** 10.1186/s12865-016-0169-5

**Published:** 2016-09-29

**Authors:** J. F. Viallard, P. Agape, V. Barlogis, G. Cozon, C. Faure, F. Fouyssac, C. Gaud, M. P. Gourin, M. Hamidou, C. Hoarau, F. Husseini, M. Ojeda-Uribe, M. Pavic, I. Pellier, A. Perlat, N. Schleinitz, B. Slama

**Affiliations:** 1 Centre Hospitalier Universitaire Haut-Lévêque, 5, Avenue de Magellan, 33604 Pessac Cedex, France; 2 Institut de Cancerologie de l’Ouest, 11 Boulevard Jacques Monod, 44800 Saint-Herblain, France; 3 CHU de Marseille - Hôpital de la Timone, 264 rue Saint-Pierre, 13385 Marseille Cedex 5, France; 4 CHU Edouard Herriot, 5 place d’Arsonval, 69003 Lyon, France; 5 Centre hospitalier intercommunal de la Haute-Saône, 2 rue Heymes BP 409, 70014 Vesoul Cedex, France; 6 CHU de Nancy, 29, avenue du Maréchal de lattre de Tassigny, 54035 Nancy Cedex, France; 7 Centre hospitalier universitaire Felix Guyon, service d’immunologie clinique, 97405 Saint Denis Cedex Ile de la Reunion, France; 8 CHU Limoges, 2, avenue Martin Luther King, 87042 Limoges cedex, France; 9 CHU de Nantes - Hôtel Dieu, 1 place Alexis Ricordeau, 44093 Nantes Cedex 1, France; 10 Centre hospitalier universitaire de Tours, 37044 Tours Cedex 9, France; 11 Centre Hospitalier Hôpitaux Civils de Colmar, 39, avenue de la Liberté, 68024 Colmar cedex, France; 12 Centre Hospitalier de la région de Mulhouse & Sud Alsace, 87, avenue d’altkirch, 68051 Mulhouse CEDEX, France; 13 CHU Fleurimont, Sherbrooke, QC J1H 5N4 Canada; 14 CHU Angers, 4 rue Larrey, 49933 Angers Cedex 9, France; 15 CHU de Rennes, 16 bd de Bulgarie, 35200 Rennes, France; 16 CHU la Timone, 264 Rue Saint-Pierre, 13385 Marseille Cedex 5, France; 17 CH Avignon, 305 rue Raoul Follereau, 84000 Avignon Cedex 9, France

**Keywords:** Primary immunodeficiency, Secondary immunodeficiency, Hizentra, Subcutaneous immunoglobulin

## Abstract

**Background:**

Although Hizentra is indicated for immunoglobulin replacement therapy in patients with primary and secondary immunodeficiencies, phase III trials have focused on patients with primary immunodeficiencies. In this 9-month, real-life, prospective, non-interventional, longitudinal, multicenter study of patients with primary and secondary immunodeficiencies in France, treatment modalities (primary endpoint), efficacy, safety, tolerability, quality of life, and treatment satisfaction were evaluated using descriptive statistics.

**Results:**

Starting in January 2012, 117 patients were enrolled (99 adults, 18 children). Secondary immunodeficiencies were present in 48.7 % of patients. At follow-up, injections were administered every 7 days in 92.2 % of patients. Nine patients (7.8 %) were taking Hizentra every 10–14 days. The median dose of Hizentra administered was 0.1 g/kg/injection. Fifty-six patients were administered doses <0.1 g/kg/injection and 13 patients were administered doses >0.2 g/kg/injection. Mean trough IgG titers were 9.0 ± 3.3 g/L (median 8.3 g/L). The mean yearly rate of infection was 1.2 ± 1.9. Mean scores on the Short Form-36 physical and mental component summaries were 46.3 ± 10.0 and 46.6 ± 9.3, respectively. Scores on the Treatment Satisfaction Questionnaire for Medication ranged from 69.9 ± 19.9 to 88.3 ± 21.2 depending on the domain. Treatment with Hizentra was well tolerated. No single drug-related systemic reaction occurred in more than one patient and few local reactions were reported (*n* = 5).

**Conclusions:**

Under real-life conditions and in a cohort that included patients with primary and secondary immunodeficiencies, treatment with Hizentra was effective and well tolerated and patients were generally satisfied with the treatment.

## Background

Subcutaneous immunoglobulin (SCIg) therapies have replaced intravenous immunoglobulin therapies in a large number of patients suffering from primary and secondary immunodeficiencies. Compared with intravenous therapies, SCIg offer the convenience of self-administration and home therapy and are associated with improved quality of life and lower cost [1–4]. Most patients express a preference for home therapy and/or subcutaneous injections [1, 5]. In children, for example, subcutaneous home therapy has been shown to be more valued because it has less impact on school, social, and family environments than intravenous injections [2].

As the volume that can be injected at any given site and the rate of injection is limited, the subcutaneous formulation is accompanied with an increase in frequency of injections. Hizentra® (CSL Behring, King of Prussia, Pennsylvania, United States of America [USA]) is a 20 % proline-based solution that is typically injected once a week in order to reach 0.4–0.8 g/kg per month. It has been shown to be efficacious and well tolerated in patients with primary immunodeficiencies [6–8]. Compared to its predecessor, Vivaglobin® (CSL Behring, King of Prussia, Pennsylvania, USA) a 16 % solution, Hizentra can be infused at a higher rate with a smaller volume. Since the commercialization of Hizentra, Vivaglobin has been discontinued in many countries including the USA (April 2011) and France (December 2013).

Although Hizentra is indicated for immunoglobulin replacement therapy in patients with primary and secondary immunodeficiencies, phase III trials were only performed in patients with primary immunodeficiencies. Few post-marketing studies have documented efficacy and tolerability under real-life conditions. To acquire a better understanding of treatment with Hizentra in France, we performed a longitudinal, observational study of treatment modalities, efficacy, safety, tolerability, quality of life, and treatment satisfaction.

## Methods

This 9-month, prospective, non-interventional, longitudinal, multicenter study was designed to describe treatment with Hizentra in France under real-life conditions. As the study was non-interventional, no specifications about treatment modalities and treatment decisions were made. Consecutive adult and pediatric patients were included if they were seen during a hospital-based consultation for primary or secondary immunodeficiency and if they were initiating treatment with Hizentra or having their Hizentra treatment modified. Patients were excluded if they were pregnant, had participated in another trial in the same domain in the previous month, or if the physician did not think that adequate follow-up would be possible. The study met the ethical standards put forth by the “French Natrional Council of the Medical Association” and the “National Commission on Informatics and Liberties”. All patients were informed with written information and gave their oral consent.

### Efficacy and safety variables

The primary end points were modalities of treatment (dose and rhythm of injections). The secondary endpoints were immunoglobulin levels, number of infections, progression of the immunodeficiency, health-related quality of life, treatment satisfaction, adverse events, and serious adverse events. Data were collected for all variables at baseline and 9 months (follow-up) except for treatment satisfaction which was only collected at follow-up.

Health-related quality of life was measured using the validated 8-domain Short Form-36 (SF-36) questionnaire [9]. Data were synthesized into the physical component summary which includes 4 domains (physical functioning, physical role functioning, bodily pain, and general health perceptions) and the mental component summary which includes the other 4 domains (vitality, social role functioning, emotional role functioning, and mental health) [10]. Patient satisfaction with treatment was measured using the validated Treatment Satisfaction Questionnaire for Medication (TSQM) which includes domains for effectiveness, side effects, convenience, and overall satisfaction [11]. Both measures are self-reported patient questionnaires with scores ranging from 0 to 100 and with higher scores signifying better health-related quality of life/treatment satisfaction.

### Statistical analysis

The Hizentra analysis group was made up of all patients who met inclusion and exclusion criteria and were treated with Hizentra. Data were analyzed using SAS® version 8 (SAS Institute Inc., Cary, North Carolina, USA). For quantitative variables, means and standard deviations (SD), medians, and ranges were calculated. For qualitative variables, number of patients and percentage of patients were calculated using the total number of patients with data available for that variable. Prospective power calculations determined that in order for subanalyses to be performed for the primary variables, an analyzable cohort of 125 patients per variable was needed. To reach this target considering an estimated 15 % loss to follow-up and non-response to questions, 150 patients needed to be enrolled.

## Results

Between January and June 2012, 117 patients were included. Ninety-nine patients were adults and 18 patients were children, 14 of which were in elementary school (Table 1). Mean age was 52.0 ± 23.9 years. Few patients (25.6 %) were employed. Approximately half of the patients (51.3 %) had a primary immunodeficiency and in 22 % of patients immunodeficiency was progressing. At inclusion, most patients (*n* = 103; 88.0 %) were being treated with an immunoglobulin and 81.6 % of these 103 patients were being treated with Vivaglobin.

**Table 1:** Patient and disease characteristics at baseline

	Hizentra N = 117
Patient characteristics	117
Male gender, n (%)	61 (52.1 %)
Age (years), N	117
Mean ± SD	52.0 ± 23.9
Median [range]	58.0 (2.0–90.0)
Weight (kg), N	115
Mean ± SD	63.0 ± 20.6
Median [range]	63.0 (11.0–115.0)
Body mass index (kg/m^2^), N	110
Mean ± SD	23.4 ± 5.0
Median [range]	23.4 (13.6–33.8)
Socio-professional status, N	117
Child in elementary school, n (%)	14 (12.0 %)
Child in middle/high school, n (%)	4 (3.4 %)
Not working^a^, n (%)	63 (53.8 %)
Adult employed, n (%)	30 (25.6 %)
Other, n (%)	6 (5.1 %)
Diagnosis, N	117
Primary immunodeficiency, n (%)	60 (51.3 %)
Secondary immunodeficiency, n (%)	57 (48.7 %)
Myeloma, n (%)	13 (22.8 %)
Chronic lymphocytic leukemia, n (%)	18 (31.6 %)
Other, n (%)	26 (45.6 %)
Duration of immunodeficiency (years), N	114
Mean ± SD	5.8 ± 5.9
Median [range]	4.3 [0.1;36.9]
Status of immunodeficiency, N	49
Progression, n (%)	11 (22.4 %)

^a^Includes sick leave, retirement, unemployment. *SD* standard deviation

The decision to treat a patient with Hizentra (Table 2) was most often informed by physician and department experience (for 71.8 % of patients), ease of administration (for 68.4 % of patients), volume of injection (for 65.0 % of patients), patient valuing independence (for 60.7 % of patients), and last gammaglobulin titer (for 57.3 % of patients).

**Table 2:** Criteria driving therapeutic decisions at baseline

Criteria driving therapeutic decisions at baseline	Hizentra N = 117
Disease-related	
Type of infectious agent, n (%)	51 (43.6 %)
Neutropenia, n (%)	11 (9.4 %)
Stage of disease, n (%)	39 (33.3 %)
Last gammaglobulin titer, n (%)	67 (57.3 %)
Other, n (%)	21 (17.9 %)
Treatment-related	
Ease of administration, n (%)	80 (68.4 %)
Volume of injection, n (%)	76 (65.0 %)
Other, n (%)	8 (6.8 %)
Patient-related	
Poor venous access, n (%)	24 (20.5 %)
Poor tolerance to IVs, n (%)	9 (7.7 %)
Difficult hospital access, n (%)	38 (32.5 %)
Busy schedule, n (%)	23 (19.7 %)
Importance of being independent, n (%)	71 (60.7 %)
Associated comorbidities, n (%)	15 (12.8 %)
Age, n (%)	42 (35.9 %)
Recurrence of infection over the previous 12 months, n (%)	26 (22.2 %)
Recurrence of infection over the previous 3 years, n (%)	24 (20.5 %)
Satisfactory renal function, n (%)	13 (11.1 %)
Other, n (%)	5 (4.3 %)
Additional considerations	
Physician and department experience	84 (71.8 %)
Staff decision	24 (20.5 %)
Failure of previous prophylactic measures	20 (17.1 %)
Other	7 (6.0 %)

*IV* intravenous therapy

At baseline, the median dose was 0.1 g/kg/injection. At follow-up, the median dose administered was 0.1 g/kg/injection (Table 3). At follow-up, 56 patients were administered doses <0.1 g/kg/injection and 13 patients were administered doses >0.2 g/kg/injection.

**Table 3:** Efficacy and modalities of treatment at baseline and 9 months

	Hizentra N = 117
Dose at baseline (g/kg/injection), N	113
Mean ± SD	0.2 ± 0.9
Median [range]	0.1 [0.0;7.0]
Dose at follow-up (g/kg/injection), N	113
Mean ± SD	0.3 ± 1.0
Median [range]	0.1 [0.0;7.0]
Schedule of injections at baseline, N	115
≤ 7 days, n (%)	111 (96.5 %)
> 7 days, n (%)	4 (3.5 %)
Schedule of injections at follow-up, N	116
≤ 7 days, n (%)	107 (92.2 %)
> 7 days, n (%)	9 (7.8 %)
Trough IgG titer at baseline (g/L), N	106
Mean ± SD	9.0 ± 6.6
Median [range]	7.7 [0.1;49.5]
Trough IgG titers (g/L) at follow-up, N	95
Mean ± SD	9.0 ± 3.3
Median [range]	8.3 [3.4;24.0]
Number of infections at follow-up, N	99
Mean ± SD	1.0 ± 1.6
Median [range]	0.0 [0.0;8.0]
Yearly rate of infection, mean ± SD	1.2 ± 1.9
Worsening of immunodeficiency at follow-up, N	98
Yes, n (%)	1 (5.3 %)

*IgG* immunoglobulin G, *SD* standard deviationc

At baseline, 96.5 % of patients were receiving injections every 7 days or less and at follow-up 92.2 % of patients were receiving injections every 7 days or less (Table 3). At follow-up, 4 patients were taking Hizentra every 10 days and 5 patients were taking Hizentra every 14 days.

**Fig. 1:**
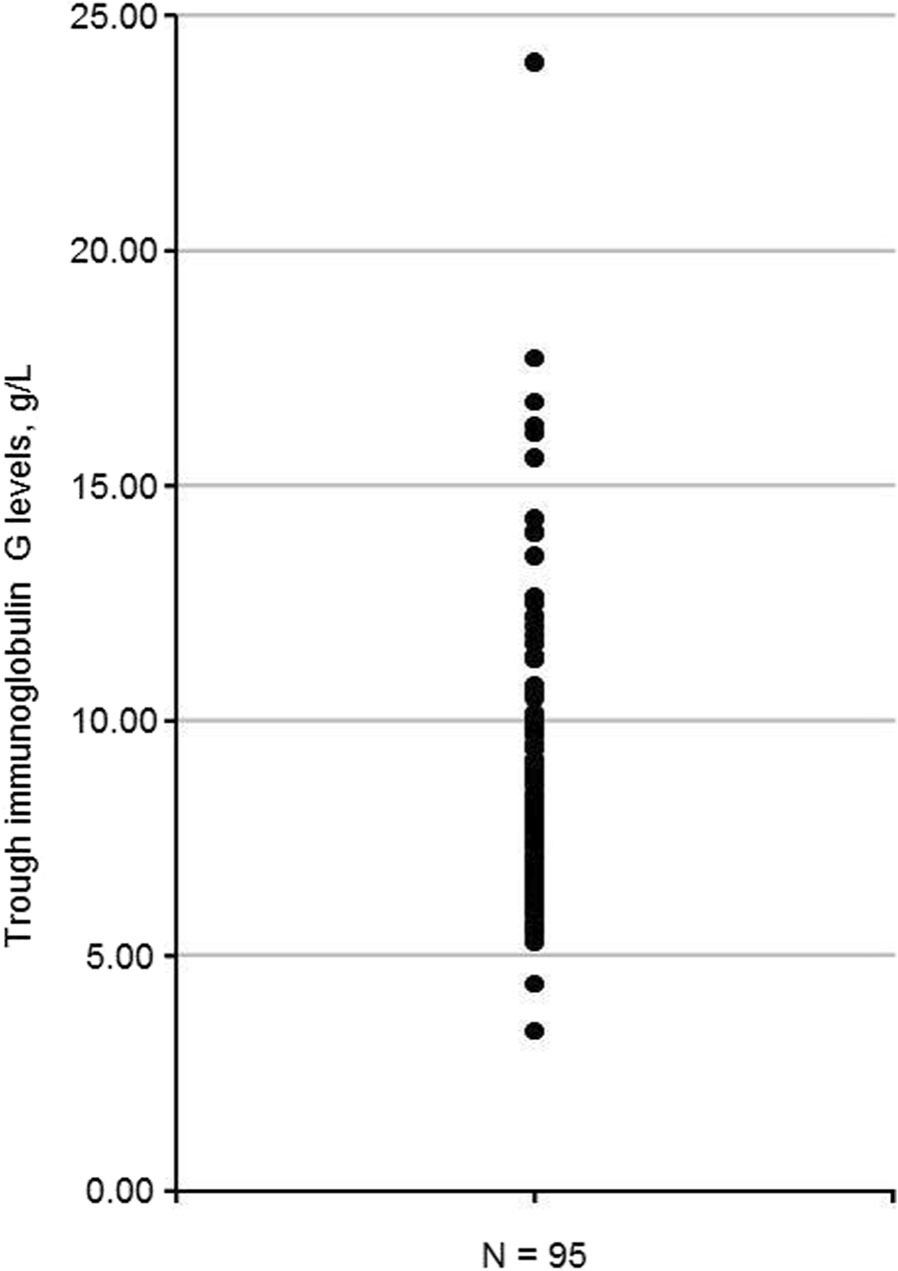
Trough immunoglobulin G levels after 9 months of Hizentra injections. Trough IgG levels at follow-up were <5 g/L in 2 patients (2.1 % of patients) and ≥10 g/L in 28 patients (29.5 % of patients). Data were available for 95 patients with primary or secondary immunodeficiencies

Mean trough immunoglobulin G (IgG) titers were 9.0 ± 6.6 g/L (median 7.7 g/L) at baseline and 9.0 ± 3.3 g/L (median 8.3 g/L) at follow-up (Table 3). Trough IgG levels at follow-up were <5 g/L in 2 patients (2.1 % of patients) and ≥10 g/L in 28 patients (29.5 % of patients) (Fig. 1). The mean yearly rate of infection was 1.2 ± 1.9 (median 0). At follow-up, 5.3 % of patients (*n* = 1) experienced a worsening of the immunodeficiency.

Mean scores on the SF-36 physical component summary were 46.4 ± 10.0 at baseline and 46.3 ± 10.0 at follow-up (Table 4). Mean scores on the SF-36 mental component summary were 45.6 ± 10.1 at baseline and 46.6 ± 9.3 at follow-up. Scores on the TSQM ranged from 69.9 ± 19.9 to 88.3 ± 21.2 depending on the domain.

**Table 4:** Quality of life and treatment satisfaction at baseline and 9 months

	Hizentra N = 117
SF-36 physical component summary	
Baseline, N	99
Mean ± SD	46.4 ± 10.0
Median [range]	48.4 [23.5;63.1]
Follow-up, N	95
Mean ± SD	46.3 ± 10.0
Median [range]	48.0 [23.6;62.6]
Change from baseline, N	92
Mean ± SD	−0.3 ± 8.9
SF-36 mental component summary	
Baseline, N	99
Mean ± SD	45.6 ± 10.1
Median [range]	46.9 [16.2;62.0]
Follow-up, N	95
Mean ± SD	46.6 ± 9.3
Median [range]	48.2 [18.8;62.8]
Change from baseline, N	92
Mean ± SD	1.1 ± 10.2
TSQM, N	94–96^a^
Effectiveness, mean ± SD	75.4 ± 14.9
Side effects, mean ± SD	88.3 ± 21.2
Convenience, mean ± SD	69.9 ± 19.9
Global satisfaction, mean ± SD	74.2 ± 19.3

^a^N varied per domain. *SD* standard deviation, *SF-36* short form 36, *TSQM* Treatment Satisfaction Questionnaire for Medication

### Safety

Nine patients (7.7 %) experienced at least 1 adverse event. Most adverse events were mild (63.6 %) or moderate (27.3 %). Most adverse events were considered possibly (72.7 %) or definitely related (18.2 %) to treatment. Treatment-related systemic reactions were headache (*n* = 1), renal colic (*n* = 1), diarrhea (*n* = 1), and sleep disturbances (*n* = 1). Local reactions at the sites of injection included pain (*n* = 2), pruritus (*n* = 1), and erythema (*n* = 2). One serious adverse event (hypertension) occurred. It was of moderate severity and considered possibly related to treatment.

## Discussion

In this real-life, non-interventional study, physicians included consecutive patients who were to be treated with Hizentra for a primary or secondary immunodeficiency. A significant number of patients with secondary immunodeficiencies (48.7 %) were enrolled. As no phase III trials have been performed in patients with secondary immunodeficiencies, this study offers a look at the modalities of treatment and the efficacy and safety of Hizentra in the broader range of patients that can be encountered in daily medical practice in France.

Treatment with Hizentra was effective. At follow-up, trough IgG levels were ≥5 g/L in 97.9 % of patients. In 29.5 % of patients, trough IgG levels were ≥10 g/L, a level which is similar to that found in healthy adults [12]. These IgG levels are consistent with data from the phase III studies in primary immunodeficiency, in which mean IgG trough levels varied from 8.1 g/L to 12.5 g/L [7, 13]. The mean yearly rate of infection herein was 1.2 ± 1.9 infections/patient/year. This rate of infection is lower than expected based rates of non-serious infections in phase III studies in primary immunodeficiency, which varied from 2.8 to 5.2 infections/patient/year [6, 7, 13], but slightly higher than that reported in a small phase IV trial (0.3 infections/patient/year) [8]. These differences amongst studies most likely reflect variations in patient populations.

Hizentra has mostly been studied using weekly injection schedules [6–8, 13]. In this study, almost all patients received Hizentra injections every seven days (97 % of patients at baseline and 92 % of patients at follow-up). In everyday life, however, the weekly schedule is considered burdensome and the question as to whether the pharmacokinetics of Hizentra are such that injections could be spaced out, has been raised. In one small study (*n* = 12), for example, in which the injection interval was 14 days, the total IgG half-life was 40.6 days and the stable trough serum IgG levels were found to be adequate (median varied from 7.24 to 7.86 g/L over 24 weeks) [14]. A small subset of patients in our study received injections every 10 to 14 days. This number increased from 3.5 % at baseline to 7.8 % at the end of the 9 months of follow-up. Additional studies would be needed to determine the characteristics of patients in whom the spacing out of injections does not compromise efficacy and tolerability.

Treatment with Hizentra was well tolerated. No single drug-related systemic reaction occurred in more than one patient and few local reactions were reported (*n* = 5). Patients reported being satisfied with the side effect profile (TSQM side effect domain score of 88). These data are also consistent with other Hizentra studies which showed that most patients experience a mild-to-moderate adverse event profile and that patients are generally satisfied with local tolerability [6, 7, 13].

Quality of life data presented herein are consistent with the fact that patients with primary and secondary immunodeficiencies generally score below the physical and mental well-being norms [5]. Mean scores on the SF-36 physical and mental summary scores were roughly 45 with maximum scores of roughly 62. No noteworthy changes in scores were noted between inclusion and follow-up. In this Hizentra cohort, 81.6 % of patients were being treated with Vivaglobin prior to inclusion, thereby suggesting that, as in other studies which specifically evaluated the switch from Vivaglobin to Hizentra [8], the transition to Hizentra was smooth and had little effect on quality of life scores.

Results of the TSQM showed that patients were generally satisfied with Hizentra treatment (mean score of 74 for global satisfaction). Scores, however, were lower than expected based on the 2015 open-label trial in which patients with primary immunodeficiencies were treated with Vivaglobin and then switched to Hizentra for 24 weeks (~90 for global satisfaction) [8]. Once again the differences in treatment populations are likely to be significant contributors to discrepancies between studies as patients with secondary immunodeficiencies are likely to be older and to have more comorbidities.

Physician and department experience, ease of administration, volume of injection, patient valuing independence, and last gammaglobulin titer were cited most frequently as having been factored into the decision to treat a patient with Hizentra. These criteria reflect known attributes of Hizentra. In particular, Hizentra, as a subcutaneous solution that can be injected at home, is easier to administer than intravenous immunoglobulins and better suited for patients who value their independence. Compared to the 16 % solution of Vivaglobin, the 20 % Hizentra solution also results in a smaller volume of injection. Although Vivaglobin is no longer commercially available in France, at the time of the study, physicians considered volume of injection as a noteworthy differentiating characteristic between Hizentra and Vivaglobin.

### Study limitations

As the current cohort did not reach sufficient power (150 patients) to perform subanalyses, additional studies in a similar patient population would be needed to understand the influence of patient characteristics and type of immunodeficiency on treatment modalities, efficacy, safety, quality of life, and treatment satisfaction.

This study did not address long-term efficacy and tolerability. However, long-term open-label extension studies have shown that short-term efficacy is maintained over time in patients with primary immunodeficiencies. In European and USA extension studies, which lasted 148 and 87 weeks, respectively, mean IgG levels were 7.97 g/L (Europe) and 11.98 g/L (USA) and the rates of infection were 3.33 infections/patient/year (Europe) and 2.38 infections/patient/year (USA) [15].

## Conclusions

Under real-life conditions and in a cohort that included patients with primary and secondary immunodeficiencies, treatment with Hizentra was effective and well tolerated; and patients were generally satisfied with the treatment.

## Abbreviations

IgG: Immunoglobulin
IV: Intravenous therapy
SCIg: Subcutaneous immunoglobulin
SD: Standard deviation
SF-36: Short form-36
TSQM: Treatment satisfaction questionnaire for medication
USA: United States of America
